# Mr. Lane on the Erect Posture

**Published:** 1909-12-04

**Authors:** 


### The Hospital

A Journal of

The Medical Sciences and Hospital Administration.

New Series. No. 145, Vol. VI. [No. 1217, Vol. XLVII.] SATURDAY, DECEMBER 4, 1909.

### MR. LANE ON THE ERECT POSTURE.

In a recent number of the *Lancet* Mr. Arbuthnot Lane lays some fresh sins to the charge, firstly, of man's upright posture, and, secondly, of present-day civilisation. Now, it is usual to describe Mr. Lane as a clinician "with ideas"; he will not plod along on Baconian principles of pure induction, but prefers the rarer, riskier, more ambitious, and much more interesting plan of collecting verifications of generalisations preformed in his own mind. His discourses have, therefore, the attractive quality of, say, Sir Almroth Wright's, and the same strong influence, too, on the rising medical generation. Of course, setting aside any invidious comparison, one cannot expect a surgeon to be as convincing in argument as a pathologist, for his experience is concerned largely with things altogether elusive of immediate demonstration; it would be as reasonable to demand mathematical certitude and philosophical or economical orthodoxy in every measure advanced by a politician. But still, Mr. Lane's writings always suggest the faculty of the investigator. One turns to his articles with as much, or even more, certainty of being interested, as in the parliamentary reports to a speech by Mr. Hilaire Belloc. And happily his latest paper has the same provocative fascination as the preceding ones.

What, to use his own words, the author wishes particularly to call attention to, is the disadvantage the individual experiences from the habit of keeping the trunk constantly erect—a habit enforced by the condition of civilisation existing at the present day. Europeans no longer recline as the ancients did, for chairs have taken the place of couches. The Anglo-Saxon, too, has given up squatting like the savage or the Bengali. And so all day long gravity is exerting an uninterrupted—an unfairly uninterrupted—downward pull on the viscera of civilised occidentals, and the bracing pressure of the front of the thighs never comes to the aid of the abdominal muscles. Even by night, and here is a fine touch of observation, there is no absolute relief, because the heavy lower part of the body sinks deeply into the soft bed. Reading this, some of the more introspective of Mr. Lane's audience may recall that when, on the links or on a country walk, they lay down to rest, their ease of body and mind was contributed to by the very material circumstance that the firm resistance of the ground, giving a comfortable hoist to the pelvis, relieved the strain on the attachments of their internal arrangements.

From this, his main thesis, Mr. Lane makes many deductions, upon which his brilliant surgical skill well entitles him to enlarge, but into which we cannot follow him here. He may hardly expect, however, quite to escape the intemperance of thought so often produced by the intoxication of a new idea which plainly has a good deal in it. Speaking of prolapse of the sigmoid flexure, he declares that "the loop formed by the sigmoid section of the large bowel falls into the pelvis and struggles with the cæcum, the transverse colon, and pelvic organs for the mastery. As the contents of the sigmoid are fairly solid, this piece of gut is a very unpleasant and obnoxious companion. Therefore, Nature endeavours to fix it in the iliac fossa. . . ." Here, besides speaking very loosely and obscurely, he is manifestly on teleological when he should be on anatomico-pathological ground. Again, the possibility is not discussed that some of the ills claimed as the effects of toxæmia, the result of constipation due to anatomical changes, may, in fact, precede both the toxic condition and the intestinal stasis, perhaps even the anatomical changes themselves, being really also manifestations, finer than these last but kindred to them, of a constitution unable to withstand modification by artificial environment. Scepticism is excusable in face of the statement that knock-knees can "materially affect length of life." There is also a curious piece of special pleading which must be mentioned. It will be remembered that in Mr. Kipling's novel, "The Light that Failed," there is a young artist in love with a lady who does not return his affection. She has a female companion with red hair who cannot understand her coldness. Both girls, as is common enough, are rather careless of their health and diet. Now on this Mr. Lane puts the following amazing interpretation: That Mr. Kipling's altogether transcendent powers of observation have led him, though ignorant of medicine, to illustrate the truth of Mr. Lane's dictum that, among the many ill-effects of chronic constipation, which somehow fall lightly on red-haired people, is to be found lack of sexual feeling. We think there is a simpler and less naïvely complimentary explanation. As is well known, a common opinion among the laity is to the effect that red hair implies an ardent disposition; and, whatever the artistic intention was in portraying unresponsiveness, clearly juxtaposition of a foil is a good way to make it evident. Or let us make an incursion into fiction after the manner of Mr. Lane, but with a different result. In *Le Père Goriot*, Rastignac and Vautrin are fellow-lodgers at a boarding house, where the food is anything but good; their lives, too, are not of the most regular. The former is dark, his companion has red hair. Rastignac thinks of little else but flames and darts; Vautrin has nothing to do with the sex, and owes his downfall to his making fun of a woman. So Balzac—wonderful man!—anticipated our discovery that intestinal stasis inhibits sexual feeling in persons of a rufous complexion. All this is nonsense, of course, but it is rather like Mr. Lane's argument. But it would be hard to find further fault with Mr. Lane's clever paper, and most presumptuous and ungrateful to try to do so.

